# Genomic Stability of Composite SCC*mec* ACME and COMER-Like Genetic Elements in *Staphylococcus epidermidis* Correlates With Rate of Excision

**DOI:** 10.3389/fmicb.2020.00166

**Published:** 2020-02-12

**Authors:** Nada Almebairik, Roxana Zamudio, Corinne Ironside, Chaitanya Joshi, Joseph D. Ralph, Adam P. Roberts, Ian M. Gould, Julie A. Morrissey, Karolin Hijazi, Marco R. Oggioni

**Affiliations:** ^1^Department of Genetics and Genome Biology, University of Leicester, Leicester, United Kingdom; ^2^School of Medicine, Medical Sciences and Nutrition, University of Aberdeen, Aberdeen, United Kingdom; ^3^Department of Tropical Disease Biology, Liverpool School of Tropical Medicine, Liverpool, United Kingdom

**Keywords:** *Staphylococcus epidermidis*, mobile genetic element, ACME, COMER, SCC*mec*

## Abstract

The epidemiological success of methicillin-resistant *Staphylococcus aureus* USA300 has been associated with the presence of two mobile elements, the arginine catabolic mobile element (ACME) and the copper and mercury resistance (COMER) element. These two mobile elements are associated with resistance to copper, which has been related to host fitness and survival within macrophages. Several studies found that ACME is more prevalent, and exhibits greater diversity, in *Staphylococcus epidermidis* while COMER has not been identified in *S. epidermidis* or any other staphylococcal species. We aimed in this study to evaluate the presence and diversity of ACME and COMER-like elements in our *S. epidermidis* clinical isolates. The genomes of 58 *S. epidermidis* clinical isolates, collected between 2009 and 2018 in a Scottish hospital, were sequenced. A core-genome phylogenetic tree and genome based MLST typing showed that more than half of the isolates belong to the clinically predominant sequence type2 (ST2) and these isolates have been found to split into two lineages within the phylogenetic tree. Analysis showed the presence of SCC*mec* in the majority of isolates. Comparative analysis identified a cluster of ACME-positive isolates with most of them belonging to ST48. ACME showed high variation even between isolates of the same ACME type and ST. COMER-like elements have been identified in one of the two major hospital adapted drug resistant ST2 lineages; and showed high stability. This difference in stability at the genomic level correlates well with the up to one hundred times higher excision frequency found for the SCC*mec* elements in ACME-containing isolates compared to COMER-like element containing isolates. ACME/COMER-like element positive isolates did not show a significant phenotype of decreased copper susceptibility, while resistance to mercury was over-represented in COMER-like element positive isolates. To the best of our knowledge, this is the first molecular characterization of COMER-like elements in *S. epidermidis* isolates. The presence of the COMER-like elements is the most prominent accessory genome feature of these successful lineages suggesting that this chromosomal island contributes to the success and wide clinical distribution of ST2 *S. epidermidis*.

## Introduction

*Staphylococcus epidermidis* and other coagulase negative staphylococci are a leading cause of nosocomial bloodstream and skin and soft tissue infections world-wide (Otto, 2009). Phylogenetic analyses have identified within the species *S. epidermidis* three major phylogenetic groups of hospital adapted isolates, two of ST2 and one of ST23 (Lee et al., 2018; Méric et al., 2018; Espadinha et al., 2019). The evolutionary pressure of the main nosocomial lineages is thought to be related to the hospital environment, but other evidence suggests that initial core genome changes in sequence type 2 (ST2) isolates result in increased acquisition of resistance traits (Lee et al., 2018). The important role of horizontal gene transfer in the evolution of *S. epidermidis* (Méric et al., 2018) and the well-established role of *Staphylococcus aureus* chromosomal cassette elements in bacterial fitness (Purves et al., 2018; Zapotoczna et al., 2018; Rosario-Cruz et al., 2019), suggest that the stepwise evolution of the cassette chromosomal elements and *mecA* gene is critical to spread of staphylococcal resistance, thus leading to the MRSA and MRSE epidemic (Rolo et al., 2017). The fine balance between acquisition and maintenance of accessory chromosomal elements shapes the evolutionary fitness of *S. epidermidis* (Zamudio et al., 2019).

The arginine catabolic mobile element (ACME) is a genomic island that first described in *Staphylococcus aureus* (MRSA) clone USA300 North American epidemic (USA300-NAE) and in *S. epidermidis* ATCC12228 (Diep et al., 2006; Planet et al., 2015). ACME is thought to increase the fitness of staphylococcus species by enhancing their ability to colonize skin and mucous membranes (Diep et al., 2008). ACME shows similar characteristics to the staphylococcal cassette chromosome *mec* (SCC*mec*) element in that it integrates into the staphylococcal chromosome at the attachment site *attB*, which is flanked by direct repeat sequences and is mobilized by the SCC*mec* encoded *ccrAB* genes. Moreover, ACME is known to commonly form a composite island with the SCC*mec* or SCC-associated genes (Diep et al., 2006; Planet et al., 2013; Lindgren et al., 2014; O’Connor et al., 2018a). To date, five distinct ACME types have been described in *S. epidermidis*; type I harbors the *arc* and *opp3* operons, type II harbors the *arc* operon only, type III harbors the *opp3* operon only, type IV harbors the *arc* and the *kdp* operons, and type V harbors all three *arc, opp3*, and *kdp* operons. The three characteristic gene clusters; *arc, opp3*, and *kdp* encode an arginine deaminase pathway, an oligopeptide permease ABC transporter and potassium ABC transporter respectively. Type IV and V ACME have been identified in oral *S. epidermidis* only (Diep et al., 2006; O’Connor et al., 2018a, b).

The copper and mercury resistance (COMER) mobile element was described for the first time in *S. aureus* (MRSA) strain USA300 South American epidemic (USA300-SAE) (Planet et al., 2015). This mobile element located in the USA300-SAE chromosome appears to replace ACME in USA300-NAE adjacent to the SCC*mec* type IV and it is thought to play a role in COMER which increases fitness as a result of increased survival to copper-mediated killing in macrophages (Planet et al., 2015; Purves et al., 2018; Zapotoczna et al., 2018). The COMER mobile element is associated with an abortive phage infection system and two main gene clusters, the *mer* operon composed of the *merR/A/B* genes and the *cop* operon composed of the *copB/L/mco* genes (Planet et al., 2015; Purves et al., 2018). To date, the COMER mobile element has not been detected in *S. epidermidis* strains.

This study investigated the prevalence and evolution of ACME and COMER mobile elements in 58 *S. epidermidis* clinical isolates retrieved from blood samples. ACME and COMER were detected and characterized using whole genome sequencing (WGS) analysis. This analysis revealed the presence of several variations of ACME as well as detection of previously undescribed COMER-like element in 11 *S. epidermidis* isolates. The excision and circularization of the ACME and COMER-like elements was investigated.

## Materials and Methods

### Bacterial Isolates

A collection of 58 *S. epidermidis* clinical isolates collected between 2009 and 2018 from blood cultures of patients admitted to the ICU of Aberdeen Royal Infirmary (Aberdeen, United Kingdom) were investigated in this study (Table 1). Twenty-five of these isolates were analyzed previously for their biocide resistance gene content (Hijazi et al., 2016). The *S. epidermidis* strains were stored at −80°C in tryptic soy broth (TSB, BD, France) with 50% glycerol (Thermo Fisher Scientific, United Kingdom). The strains were streaked on tryptic soy agar (TSA) plates (prepared by adding 1.5% agarose to TSB) and incubated at 37°C for 16–24 h. Single colonies were then sub-cultured in TSB and incubated overnight at 37°C/200 rpm in a shaking incubator (Innova 4000, New Brunswick Scientific, United Kingdom).

**TABLE 1:** *S. epidermidis* isolates.

	Isolate	Accession	Date	MLST	COMER-like	ACME	SCC*mec* type
NB22338Q	STAPH 48	SAMN12840226	July 2009	ST559			SCC*mec*-IV
NB23267V	STAPH 49	SAMN12840227	July 2009	ST2	COMER		SCC*mec*-III
NB41003Z	STAPH 51	SAMN12840228	May 2010	ST83			SCC*mec*-IV
NB14428Z	STAPH 53	SAMN12840229	May 2010	ST5		ACME-V	SCC*mec*-IV
NB21991B	STAPH 54	SAMN12840230	July 2010	ST5			SCC*mec*-IV
NB24551C	STAPH 56	SAMN12840231	August 2010	ST2			SCC*mec*-IV
NB06465P	STAPH 58	SAMN12840232	March 2011	ST2			SCC*mec*-IV
NB12208Q	STAPH 59	SAMN12840233	April 2011	ST83			SCC*mec*-IV
NB24506S	STAPH 60	SAMN12840234	August 2011	ST2	COMER		SCC*mec*-III
NB25777X	STAPH 61	SAMN12840235	September 2011	ST2	COMER		SCC*mec*-III
NB26250A	STAPH 62	SAMN12840236	September 2011	ST2	COMER		SCC*mec*-III
NB26865Y	STAPH 63	SAMN12840237	September 2011	ST2	COMER		SCC*mec*-III
NB28476A	STAPH 64	SAMN12840238	October 2011	ST2	COMER		SCC*mec*-III
NB13883R	STAPH 66	SAMN12840239	April 2012	ST19			
NB25117Z	STAPH 67	SAMN12840240	July 2012	ST210			
NB25100R	STAPH 68	SAMN12840241	July 2012	ST54		ACME-I	
NB29474S	STAPH 69	SAMN12840242	September 2012	ST2			SCC*mec*-IV
NB41238E	STAPH 70	SAMN12840243	December 2012	ST2			SCC*mec*-IV
NB01101D	STAPH 73	SAMN12840244	January 2013	ST204			
NB02243Y	STAPH 74	SAMN12840245	January 2013	ST470		ACME-IV	
NB03947K	STAPH 75	SAMN12840246	January 2013	ST2	COMER		SCC*mec*-III
NB22235H	STAPH 77	SAMN12840247	January 2013	ST59			SCC*mec*-IV
NB26073F	STAPH 78	SAMN12840248	July 2013	ST2	COMER		SCC*mec*-III
NB33101Z	STAPH 79	SAMN12840249	September 2013	ST48		ACME-I	SCC*mec*-IV
NB671022	STAPH 83	SAMN12840250	February 2014	ST2			SCC*mec*-IV
NB03169X	STAPH 1	SAMN12840193	January 2016	ST2	COMER		SCC*mec*-III
NB03186T	STAPH 2	SAMN12840194	January 2016	ST35		ACME-I	SCC*mec*-IV
NB09909V	STAPH 3	SAMN12840195	March 2016	ST48		ACME-I	SCC*mec*-IV
NB10503A_1	STAPH 4	SAMN12840196	March 2016	ST59			SCC*mec*-IV
NB10503A_2	STAPH 5	SAMN12840197	March 2016	ST5			SCC*mec*-IV
NB11089T	STAPH 6	SAMN12840198	March 2016	ST5			SCC*mec*-IV
NB11582T	STAPH 7	SAMN12840199	April 2016	ST2			SCC*mec*-IV
NB12943Y	STAPH 8	SAMN12840200	April 2016	ST2	COMER		SCC*mec*-III
NB13468Z	STAPH 9	SAMN12840201	April 2016	ST23			SCC*mec*-IX
NB13483J	STAPH 10	SAMN12840202	April 2016	ST2	COMER		SCC*mec*-III
NB17762B	STAPH 12	SAMN12840203	May 2016	ST528		ACME-I	
NB18619Q	STAPH 13	SAMN12840204	June 2016	ST2			SCC*mec*-IV
NB20483A	STAPH 14	SAMN12840205	June 2016	ST2			SCC*mec*-IV
NB21362A	STAPH 15	SAMN12840206	June 2016	ST2			SCC*mec*-IV
NB24986M	STAPH 16	SAMN12840207	July 2017	ST54		ACME-I	SCC*mec*-IV
NB24987J	STAPH 17	SAMN12840208	July 2017	ST210		ACME-IV	SCC*mec*-IV
NB31156H	STAPH 18	SAMN12840209	September 2017	ST65			
NB31437E	STAPH 19	SAMN12840210	September 2017	ST73		ACME-II	
NB34495N	STAPH 20	SAMN12840211	October 2017	ST2			SCC*mec*-IV
NB36056Y	STAPH 21	SAMN12840212	October 2017	ST2			SCC*mec*-IV
NB36246W	STAPH 22	SAMN12840213	October 2017	ST210		ACME-IV	SCC*mec*-V
NB37325D	STAPH 23	SAMN12840214	October 2017	ST2			SCC*mec*-IV
NB37754V_1	STAPH 24	SAMN12840215	October 2017	ST2			SCC*mec*-IV
NB37754V_2	STAPH 25	SAMN12840216	October 2017	ST591			SCC*mec*-IV
NB38801E	STAPH 27	SAMN12840217	November 2017	ST2			SCC*mec*-IV
NB40693L	STAPH 28	SAMN12840218	November 2017	ST2			SCC*mec*-IV
NB41771A	STAPH 29	SAMN12840219	November 2017	ST2			SCC*mec*-IV
NB42838N	STAPH 30	SAMN12840220	December 2017	ST2			SCC*mec*-V
NB43408J	STAPH 31	SAMN12840221	December 2017	ST2			SCC*mec*-V
NB03117S	STAPH 32	SAMN12840222	January 2018	ST2			SCC*mec*-IV
NB03149G	STAPH 33	SAMN12840223	January 2018	ST2			SCC*mec*-IV
NB05012Y	STAPH 34	SAMN12840224	January 2018	ST48		ACME-I	SCC*mec*-IV
NB05684Z	STAPH 35	SAMN12840225	February 2018	ST48		ACME-I	SCC*mec*-IV

### Whole Genome Sequencing

Genomic DNA extraction was performed using NucleoSpin Tissue Kit (Macherey-Nagel, Germany) following the manufacturer’s instructions. Genomic DNA was eluted in 30 μl pure water, and the concentration and purity measured by the NanoDrop 2000c spectrophotometer (Thermo Fisher Scientific, United Kingdom). Genomic DNA was sequenced on a HiSeq 4000 sequencer (Illumina, The Wellcome Trust Centre for Human Genetics at the University of Oxford) with 150 bp paired-end reads. Genome sequences were trimmed using Trimmomatic (Bolger et al., 2014), then assembled using SPAdes software version v3.9.0 (Nurk et al., 2013) and quality-assessed with QUAST (Gurevich et al., 2013). The draft genomes in contig level for all 58 isolates were submitted to the GenBank under the project number PRJNA574294, the GenBank accession number for the isolates is shown in Table 1.

### Multilocus Sequence Typing (MLST) and Core-Genome Phylogeny Tree

The sequence type (ST) of each isolate was specified by submitting the whole genome sequence (WGS) to the *S. epidermidis* MLST 2.0 online database (Larsen et al., 2012). The core-genome alignment was generated using Roary software version 3.11.2 (Page et al., 2015). Then a maximum likelihood tree for 58 Aberdeen isolates and the BPH0662 reference genome was build using RAxML software version 8.2.X (Stamatakis, 2014). Rstudio v 1.1.453^1^ and the R package ggtree v 1.13.5 (Yu et al., 2017) was used for visualization and annotation of the phylogeny tree.

Maximum likelihood tree for the 58 Aberdeen isolates and 225 isolates from Lee’s collection (NCBI BioProject numbers: PRJEB12090, PRJNA470534, and PRJNA470752) (Lee et al., 2018) and the BPH0662 reference genome (10.1099/mgen.0.000077) was generated through the iqtree tool (doi: 10.1093/molbev/msu300) by using the Hasegawa–Kishino–Yano model and gamma distribution (Yang, 1994) (10.1007/bf00160154).

### Identification of SCC*mec*, ACME, and COMER-Like Elements

For each isolate, the presence and the type of the SCC*mec* mobile element was determined by submitting the genome sequence to the SCC*mec*Finder 1.2 online database (Kaya et al., 2018). Contigs were aligned to the ACME and COMER elements previously characterized in *S. aureus* USA300 strain FPR3757 (GenBank accession number CP000255) and strain CA12 (GenBank accession number CP007672) respectively using the BLAST software (AltschuP et al., 1990) in order to detect the presence of ACME and COMER elements in the study isolates. In some isolates the ACME and COMER element-associated genes were identified on three and five separate contigs – in this case ACME-I and COMER elements were assembled by PCR. These contigs were organized, reoriented and assembled using BLAST software, Reverse complement tool^2^ and ISfinder online database (Siguier, 2006). For further investigations, contigs were annotated using the RAST server (Aziz et al., 2008), Artemis sequence viewer (Berriman, 2003) and SnapGene viewer (GSL Biotech)^3^. This is followed by multiple alignment of the ACME and COMER mobile elements using Mauve software to detect the variation in these elements among the isolates (Darling, 2004).

### Metal Susceptibility

The susceptibility of these isolates to several metals including copper sulfate (CuSO_4_), nickel sulfate (Niso_4_), iron chloride (FeCl_3_), manganese sulfate (MnSO_4_), zinc sulfate (ZnSO_4_), cobalt bromide (CoBr_2_), cadmium chloride (CdCL_2_), mercuric chloride (HgCl_2_), and sodium arsenate (AsNA_2_) (all Sigma) was determined using disc diffusion testing. Several colonies from overnight TSA cultures were collected with a sterile loop and re-suspended into Mueller Hinton broth (Oxoid, United Kingdom) to OD_600_ = 0.5. A Mueller Hinton agar (MHA) plate was seeded with the culture using a sterile swab in three different directions to obtain uniform growth. Ten microliter of 1M solutions of each metal were loaded into a sterile filter discs, obtained from filter papers (Munktell, United Kingdom), and allowed to dry for 15 min. Filter discs were then firmly applied to the surface of the MHA plate and incubated overnight at 37°C. The metal susceptibility was determined using the epidemiological cut-off values (ECOFF) (Morrissey et al., 2014) followed by Fisher’s Exact test to find the susceptibility association among the isolates^4^. A linear regression model was applied to predict the disk diffusion values based on the presence or absence of the composite element by using the lm function from stats’s R package.

### Site-Specific Excision of SCC*mec*, ACME, and COMER-Like Elements

A qPCR-based method was used to quantify the rate of excision and circularization of the composite SCC*mec* and ACME/COMER-like elements. Specific primers were designed using SnapGene viewer software (Table 2) and used to amplify the element attachment sites, the circularized SCC*mec* and SCC*mec* plus the ACME/COMER-like element and the excision products (chromosome junction after the element excision). Excision of the SCC*mec* and ACME/COMER-like element was induced in selected isolates using 0.5 μg/ml Mitomycin C (Sigma-Aldrich). qPCR reactions were performed using the TaqMan Fast Advanced Master Mix (Thermo Fisher Scientific) with the following run protocol: initial denaturation step at 95°C for 20 s then 45 cycles of 95°C for 3 s and 60°C for 30 s. The reactions were carried out in MiroAmp Fast 96-Well Reaction Plate (Applied biosystems) using a 7500 real-time PCR machine (Applied Biosystems). The rate of excision was measured using qPCR by comparing the excised circularized element and the reconstituted chromosomal attachment site to the integrated element using this formula: 2^–Δ^*^*CT*^* (Livak and Schmittgen, 2001).

**TABLE 2:** Primers and probes used in the study.

Primers	Sequence	Annealing temperature (°C)
**SCC*mec*-IV AND ACME PRIMERS**		
qAttR1 fw	TACGCTCTATCATTCAGCACTATGA	55
qAttR1 rev	ATGATAAGCTTCTTAAAAACATAACAGC	55
Probe R1	CACCAAATGATGCGCGTCATATTGATAGA	61
qAttR2 fw	AAACCAAAGTCGATATCATCATTTTG	55
qAttR2 rev	CAGCATTATCGTAAGTTAACTACATT	55
Probe R2	CGACTTTAATTATAAAAAACCGCACTCTTAACCG	61
qAttL fw	GAAGTGATTTTACGATATCACCTTCT	55
qAttL rev	CATTTAAGATGGAATTCAAATTTTATTTTACTTTC	54
Probe L	TATATTTCAGTAGGCACCGACGTATACAGAATCA	61
**SCC*mec*-III AND COMER-LIKE PRIMERS**		
qAttR1 fw	CTCTCTTAAATTTTTGTTTGATTAGATTAGACC	56
qAttR1 rev	ACTTGAAATGAAAGACTGCGG	56
Probe R1	ACGCATTCAATATGTCTACACGTGAATTTAGTCT	62
qAttR2 fw	ACACCAGCTTTCTATGTAGGTAA	55
qAttR2 rev	ATTTTATTGGAGATACTATATACTTAACCAATTTC	54
Probe R2	GCGAAGAAAGCCATTATTATGAGGTGATTGTAG	61
qAttL fw	GAGCGACAAATTCCAATGTAATAGTA	55
qAttL rev	ACCAAACAATGAATATATAATACATTTAAGATG	54
Probe L	GTCGCTCTTTCGTTTCAGTTAAGGAAAATG	61

## Results

**FIGURE 1:**
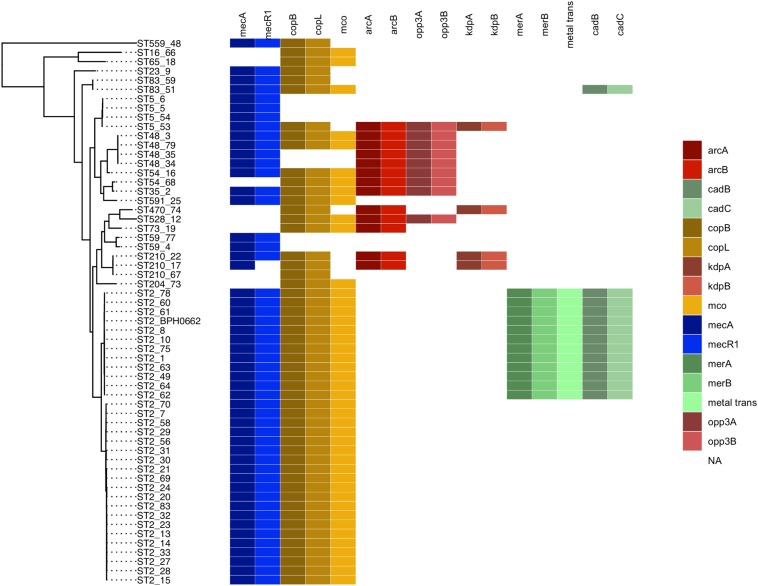
Maximum likelihood phylogenetic tree constructed using core genome alignment from the 58 *S. epidermidis* clinical isolates. The phylogenetic tree is annotated with the isolate’s sequence type ST. Colored boxes to the right of each strain name illustrated the distribution of the SCCmec (blue), cop operon (yellow), ACME (red), and COMER-like element (green) characteristic genes among the *S. epidermidis isolates. S. epidermidis* BPH0662 used as reference strain in this phylogenetic tree.

The evolution of *S. epidermidis* isolates recovered from bloodstream infections between 2009 and 2018 at Aberdeen Royal Infirmary was studied by core-genome phylogenetic analysis (Figure 1). Genome-based MLST revealed that more than 50% of the isolates (31 of 58) belong to the major hospital acquired multidrug resistant ST2 lineage which in turn was found to separate into two sub-lineages as previously described (Lee et al., 2018). The other 16 STs were distributed among different lineages in the phylogenetic tree. Four ST5 isolates, four ST48 isolates and three ST210 isolates. Of the 58 *S. epidermidis* isolates, 86% (50/58) were methicillin resistant *S. epidermidis* (MRSE) as they harbored the *mecA* gene. The vast majority of these MRSE contained SCC*mec* types III and IV (Table 1). The other eight isolates (14%) considered methicillin sensitive *S. epidermidis* (MSSE) lacked the *mecA* gene. All the isolates harbored the penicillin resistance *blaZ* gene except isolate STAPH74.

**FIGURE 2:**
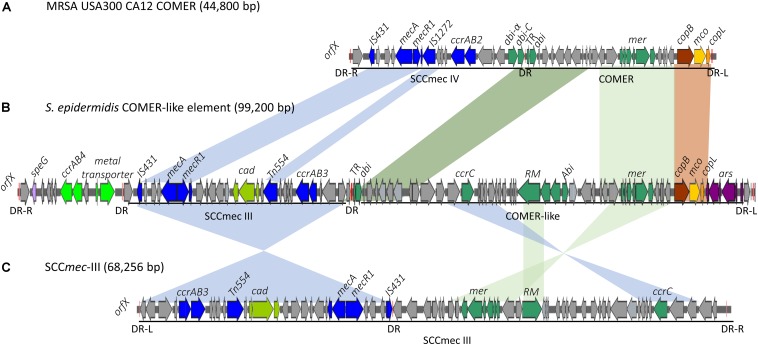
Schematic map of the COMER-like elements in *S. epidermidis*. The COMER element previously described in MRSA USA300 strain CA12 (GenBank accession number CP007672) **(A)** and the SCCmec-III element previously described in strain 85/2082 (GenBank accession number AB037671) **(C)** are included for comparison. Characteristic genes of the COMER-like element are colored including the *mer* operon, the *abi* genes, and the restriction modification system (green), the *cop* operon (yellow-brown) and ars operon (purple). The SCC*mec* element genes colored include the *mecA, ccrAB3* genes (blue), and the *cad* operon (light green). An additional module harboring a metal transporter, *ccrAB4* (bright green) and *speG* genes (light purple) is found upstream SCC*mec-*III in our *S. epidermidis* strains **(B)**. Homologous regions between the different elements are shown by shading. The direct repeats (DR) are indicated.

Eleven of the 31 isolates belonging to the clinically predominant ST2 (35%) were found to be positive for a COMER-like element (Figure 1). These eleven isolates were clustered in one of the two ST2 sub-lineages, also containing the reference isolate BPH0662 (10.1099/mgen.0.000077) (Lee et al., 2018). We have named this element COMER-like element as it harbored the *mer* and *cop* operons as well as *abi* gene that exhibited very high nucleotide sequence identity with the *mer/cop* operons (99.98% identity over 11,028 bp) and *abi* gene cluster (100% identity over 1,197 bp) located on the COMER element of MRSA USA300 strain CA12 (GenBank accession number CP007672) (Figure 2). However, additional genes not present in COMER USA300, including *ars* operon and a type I restriction modification system, were identified in the *S. epidermidis* COMER-like elements. The COMER-like element was found to be a composite island of 99.2 kb in size. The structural organization of this composite island was identical in all eleven isolates with the COMER-like element located immediately downstream of SCC*mec*-III and encoding the *mecA* gene and the *cad/mer* operons with > 99.8% nucleotide sequence identity with the corresponding genes in SCC*mec*-III of strain 85/2082 (GenBank accession number AB037671). Additionally, this composite island contained a module harboring the *speG* and *ccrAB4* genes and the gene of a putative further metal transporter (locus_tag = ″F9B38_12675) (Figure 2). In contrast to our composite *S. epidermidis* element, the COMER element in USA300 is located downstream of SCC*mec*-IV (Figure 2). This is the first description of a COMER-like element in *S. epidermidis* strains.

**FIGURE 3:**
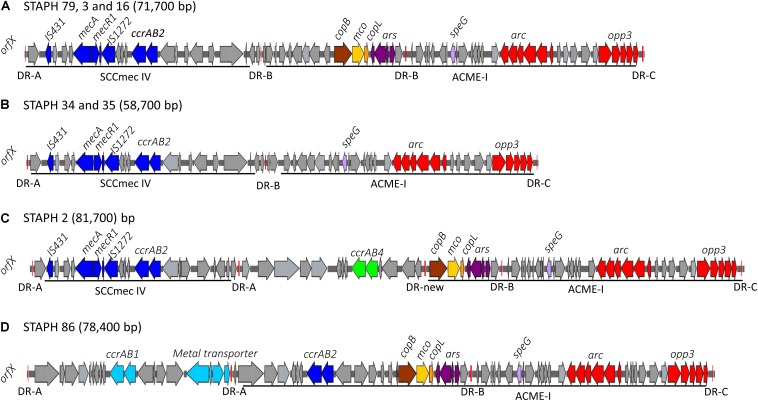
Schematic map of the ACME-I in *S. epidermidis*. comparative organization of ACME-I composite island in the seven ST48, ST54, ST35 *S. epidermidis* isolates. The size of each ACME composite island is indicated after the strain names. Four distinct ACME-I composite island were defined according to the additional modules in these elements **(A–D)**. Each gene or group of genes of interest is shaded in color; *arc* and *opp3* operons (red), *speG* (light purple), *copB* (brown), *mco* (light yellow), *copL* (ornage), *ars* operon (dark purple), *mecA, mecR1, IS431, IS1272*, and *ccrAB2* (dark blue). The direct repeat (DR) sequences DRA, DR-B, DR-C, and the new DR are indicated.

The arginine catabolic mobile element (ACME) was detected in 9/50 (18%) of the MRSE isolates and 4/8 of the MSSE isolates (Figure 1). These ACMEs encoded the *arc* and/or *opp3* and/or *kdp* operons as previously described for ACME in *S. epidermidis* (Miragaia et al., 2009; Barbier et al., 2011; Onishi et al., 2013; Soroush et al., 2016; McManus et al., 2017; O’Connor et al., 2018a). The ACME-I element was the predominant ACME type and all ACME-I containing isolates, except isolate STAPH12 (ST528), found to cluster in one lineage in the phylogenetic tree containing ST48, ST54 and ST35. The ACME-I element in these isolates was assembled and found to harbor the *arc, opp3* operons, and the *speG* gene, all flanked by 15 bp direct repeats (DR_B and DR_C). Additionally, one or more modules were found to colocalize with the ACME-I forming a composite island (Figure 3). Comparative analysis tools showed high variability of ACME-I composite island. In 3 out 7 isolates [ST48 (*n* = 2) and ST54 (*n* = 1)], the ACME-I was located downstream of the SCC*mec*-IV, separated by a module composed of *ars* and *cop* genes. This module and the SCC*mec*-IV were flanked by DR_A and B respectively at the 5′end in *orfX* (Figure 3A). In 2 out of 7 ST48 isolates the ACME-I element colocalized with SCC*mec*-IV but lacked the *ars*/*cop* gene modules (Figure 3B). In one ST35 isolate an additional module harboring the *ccrAB4* genes flanked by DR_A and DR_Q was inserted between the *ars/cop* gene modules and the SCC*mec*-IV (Figure 3C). The ACME composite island in one ST54 isolate lacked the *mecA* gene and included only two modules located upstream the *ars*/*cop* genes module and the ACME-I. These two modules were flanked by DR_A, _A, and _B respectively at the 5′end in *orfX* (Figure 3D).

**FIGURE 4:**
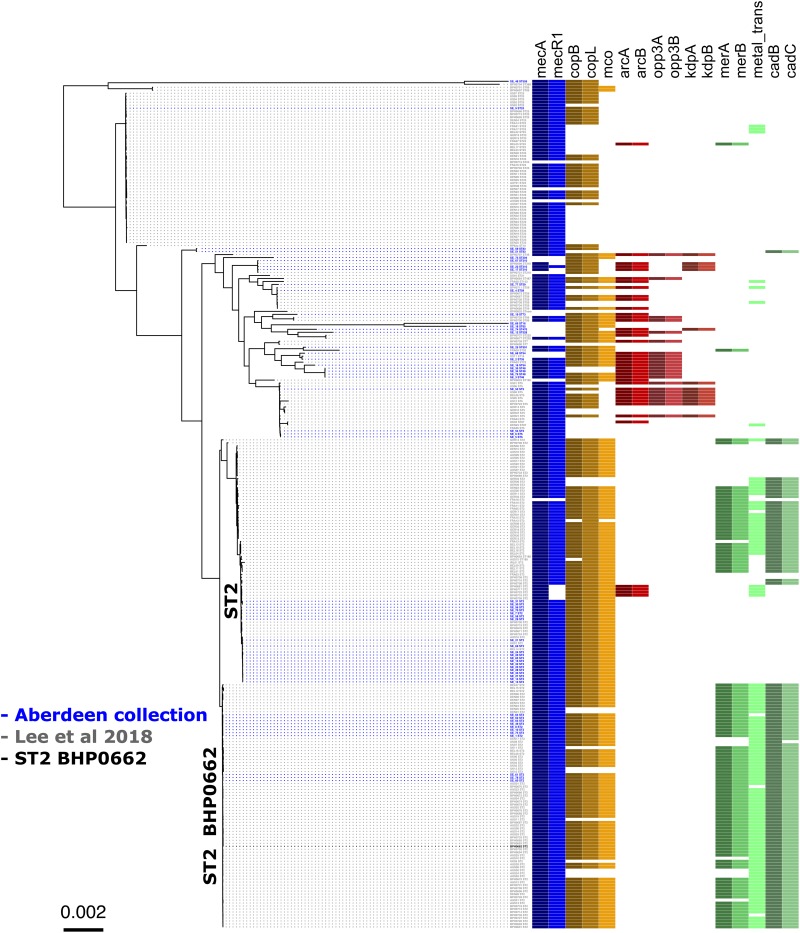
Core genome phylogenetic tree of a world-wide collection of *S. epidermidis* genomes. Maximum likelihood tree of the 58 Scottish isolates (Table 1) (isolates in blue) and the dataset from a world-wide collection of 225 isolates (isolates in gray) (NCBI BioProject numbers: PRJEB12090, PRJNA470534, and PRJNA470752) (Lee et al., 2018). As in Figure 1, the tree is annotated with the isolate’s sequence type (ST) and displays the colored boxes of the heatmap illustrating the distribution of the characteristic genes of the SCCmec (blue), cop operon (brown/yellow), ACME (red) and COMER-like element (green) among the *S. epidermidis* isolates. Isolate BPH0662 (isolate in black) is used as reference strain in this phylogenetic tree.

To validate the distribution of the composite SCC elements on a larger dataset and at global level, we generated a core genome phylogenetic tree that included a collection of 225 worldwide *S. epidermidis* genomes (Lee et al., 2018; Figure 4). The phylogenetic tree was build using the alignment of 1,898 core genes which represent 61.1% (1,706,669 bp) of the reference genome BHP662. This phylogenetic tree combining both datasets showed large clustering of ST2 isolates and their sub-lineages as found in the Scottish collection. Mapping of the key genes of our composite SCC elements, showed again exceptional stability of the COMER-like element. In the larger dataset this element mapped in all 82 isolates belonging ST2 BPH0662 sub-lineage and also partially (38/82) to a second ST2 sub-lineage of ST2 isolates (Figure 4). The mapping in addition confirmed a high variability of ACME elements and their presence in multiple ST and sub-lineages. Copper resistance genes were present in most isolates, even in absence of the COMER-like and ACME elements (Figure 4). As expected nearly all isolates carried SCC*mec* elements.

**FIGURE 5:**
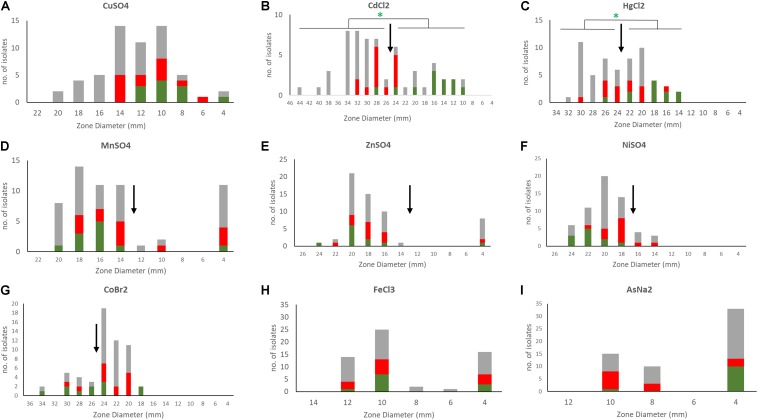
Susceptibility profiles of *S. epidermidis* to different metals. Disc diffusion inhibition zones were defined for the 58 *S. epidermidis* isolates by exposing bacteria to discs containing 10 μl of 1M metal stocks (0.2 M for mercury and arsenic) [**(A)** copper, **(B)** cadmium, **(C)** mercury, **(D)** manganese, **(E)** zinc, **(F)** nickel, **(G)** cobalt, **(H)** iron, and **(I)** arsenic]. Metals tested included copper sulfate (CuSO_4_), nickel sulfate (Niso_4_), iron chloride (FeCl_3_), manganese sulfate (MnSO_4_), zinc sulfate (ZnSO_4_), cobalt bromide (CoBr_2_), cadmium chloride (CdCL_2_), mercuric chloride (HgCl_2_), and sodium arsenate (AsNA_2_). In the bar charts ACME containing isolates are shown in red, COMER-like element containing isolates in green and all other isolates in gray. ECOFFs are shown in arrows. Statistical analysis of the association of isolates carrying COMER-like elements with cadmium and mercury susceptibility is shown (Fisher’s exact test).

A phenotypic analysis of metal susceptibility of these isolates was performed by disc diffusion susceptibility testing on our Scottish isolates (Figure 5). As no breakpoints for metal resistance exist, we determined the ECOFF (Morrissey et al., 2014). The ECOFF breakpoints, based on the normal distribution of the susceptibility, was defined using the Shapiro–Wilk test. All susceptibility data, except for copper, were not normally distributed. Still we did not define an ECOFF for iron and arsenic as the reduced inhibition zone appears to indicate intrinsic resistance of the all isolates. Isolates belonging to the ST2- BPH0662 lineage carrying the COMER-like element were found to be significantly associated with reduced susceptibility to mercury and cadmium (*p* < 0.05) using Fisher’s Exact test (Figures 5B,C). The phenotypes match to the presence of the cadmium and mercury transporter in these strains. The linear regression model was applied to predict the disk diffusion value of the cadmium and mercury based on the presence or absence of the COMER-like element in ST2 isolates, the coefficient of correlation for mercury was *R*^2^ = 0.57 and for cadmium was *R*^2^ = 0.75. No association was found for ACME-carrying isolates.

**FIGURE 6:**
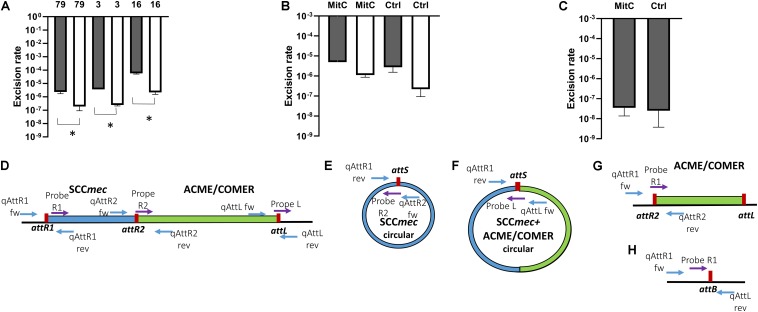
Excision rates of composite SCC*mec* elements. The rate of excision was measured using real-time qPCR by comparing the excised circularized element and the reconstituted chromosomal attachment site to the integrated element using this formula: 2^–Δ^ *^*CT*^*. The columns represent the rate of excised element, SCC*mec* alone (gray) and the composite SCC*mec*/ACME (white), in isolates STAPH79, STAPH3, and STAPH16. Error bars represent the mean SD of the excised element with and without mitomycin C, asterisk (*) indicates *p*-value smaller than 0.05 (*p* < 0.05). **(A)** Induction with mitomycin C did not affect the excision ratio of the SCC*mec* (gray) nor the composite SCC*mec*/ACME (white) in isolate STAPH79 comparing to the control (Ctrl) **(B)**. In STAPH60, like in STAPH79, the mitomycin C induction had no effect on excision frequency with respect to control (Ctrl) **(C)**. The schematic representation of the excision of SCC*mec* and ACME/COMER-like elements is shown in **(D–H)** including the chromosome containing the integrated SCC*mec* (blue) with the ACME/COMER elements (green) **(D)**, the circular form of the SCC*mec* **(E)** and circular form SCC*mec* with ACME/COMER-like element **(F)**, the chromosome with the ACME/COMER element after excision of SCC*mec* only **(G)** and the reconstituted chromosome after excision of the whole composite element **(H)**. The *att* sites are shown in red and the primers used for qPCR are shown as blue arrows and TaqMan probes as purple arrows. The maps are not drawn to scale.

The rate of excision of the composite SCC*mec* ACME and COMER-like elements was measured by quantitative real time PCR amplification of the reconstituted chromosomal attachment/target site and the circularized elements. Results were compared to the quantification of the integrated elements (considered equivalent to the total quantification of chromosomes). Results for the ACME-I isolates STAPH16, STAPH3, and STAPH79 showed that the SCC*mec*-IV element alone did excise at a frequency ranging from 5.3 × 10^–5^ (isolate 16) to 2.7 × 10^–6^ (isolate 79) (Figure 6A). The excision of the whole composite SCC*mec*-IV/ACME-I element was significantly lower (1.7 × 10^–6^ – 1.2 × 10^–7^) (*p* < 0.05) using *t*-test (Figure 6A). The ratio of excision did not significantly change (*p* > 0.05) in the presence of mitomycin C for any of the isolates, as shown for isolate STAPH79 (Figure 6B). Results for the COMER-like element in isolate STAPH60 showed that the SCC*mec*-III excised alone at a frequency equal to 2.3 × 10^–8^ and as with ACME the ratio of excision did not change with mitomycin C induction (Figure 6C). The whole composite SCC*mec*-III/COMER-like element excision was under the detection limit of our assay. The excision of the SCC*mec*-III is significantly lower than the SCC*mec*-IV excision by 100 times (*p* < 0.05) using *t*-test.

## Discussion

In recent decades, *S. epidermidis* has emerged as a nosocomial pathogen. Genomics showed that the two *S. epidermidis* hospital-adapted lineages within ST2 are nearly pan-drug resistance (Lee et al., 2018). The evolution of *S. epidermidis* from commensal to disease-causing bacteria is poorly investigated, although several studies suggested that *S. epidermidis* acts as a reservoir of resistance genes for *S. aureus* (Otto, 2009). Comparative genomic analysis of *S. epidermidis* clinical isolates in this study revealed a high prevalence of methicillin resistance and the MLST showed that more than half the isolates belonged to clinically predominant ST2 confirming the dissemination of this multidrug resistance lineage in our study isolates. The increased capacity of ST2 to acquire drug resistance phenotypes has been attributed to a single polymorphism in the core genome RNA polymerase gene *rpoB* (Lee et al., 2018). While this original SNP may well be at the origin of the success of ST2, the dominant presence of a large composite COMER-like element in the ST-2 BPH662 lineage is likely very significant to the success of the lineage. The COMER-like element was exceptionally stable when compared to other SCC composite structures, for example ACME (Miragaia et al., 2009; O’Connor et al., 2018b). In *S. aureus* USA300 the copper resistance phenotype conferred by the COMER element was found to be associated with increased macrophage resistance and fitness (Purves et al., 2018; Zapotoczna et al., 2018). In this study we observed only a mercury and cadmium resistance phenotype only in isolates bearing the COMER-like element, but this may be related to the wide distribution of the copper gene cluster among most *S. epidermidis* isolates. The role of the additional genes within the COMER-like element, and absent in USA300, in the success of the BPH662 lineage, cannot be inferred at this stage.

This investigation analysing the diversity of composite SCC elements identified, in addition to the well-known ACME elements characterized in *S. epidermidis*, a highly conserved COMER-like element in one of the main ST2 clusters, the BPH0662-lineage (Zamudio et al., 2019). Different types of ACME identified in this study, including ACME type IV and V, harbored the *kdp* operon linked previously to oronasal *S. epidermidis* isolates only (O’Connor et al., 2018b). The findings from oronasal *S. epidermidis* isolates harboring ACME suggested that each ACME type emerged from the respective identical or closely related ST rather than the site from which the isolates harboring ACME were recovered (O’Connor et al., 2018b). For example, isolates harboring ACME type II and V investigated in this study belonged to ST73 and ST5 respectively, the same STs harboring ACME types II and V in oronasal *S. epidermidis*. Isolates harboring ACME type I belonged to ST48 as well as ST54 and ST35 (a double locus variant of ST48) and isolates harboring ACME type IV belonged to ST210. However, one isolates with ACME type I and one isolate with ACME type IV belonged to ST528 and a new ST which is closely related to ST470 respectively, suggesting emergence from a different lineage. The variations in the ACME-composite island investigated in this study suggest the rapid evolution of ACME in *S. epidermidis*. Previous studies of ACME prevalence amongst *S. epidermidis* populations reported a higher prevalence of ACME in MSSE than MRSE (Miragaia et al., 2009; Barbier et al., 2011; Onishi et al., 2013). Our data are supportive of this finding as ACME was identified in 9/50 (18%) of the MRSE and in 4/8 (50%) of the MSSE.

In addition to confirm excision of the SCC*mec* elements as previously reported (Stojanov et al., 2015), we report circularization of the ACME element and the *S. epidermidis* COMER-like element. The excision frequency of the SCC*mec*-IV in ACME-I containing isolates 16, 3 and 79 ranged from 5.3 × 10^–5^ to 2.7 × 10^–6^ while the excision of the composite SCC*mec*-IV/ACME-I element was approximately 5 times lower. This finding correlates with previous studies on *S. aureus* in which the rate of excision was estimated to be lower than 10^–4^ (Ito et al., 1999; Stojanov et al., 2015). Interestingly, the net production of excision products in the presence of mitomycin C did not change for any of the isolates different to what observed previously by others (Liu et al., 2017). The SCC*mec*-III excision in COMER-like element containing isolate was up to 100 times lower than the SCC*mec*-IV excision. This shows a strong correlation of excision frequency and genomic variability. We hypothesize that this difference in excision of both the SCC*mec* and of the composite elements may explain the high genomic stability of the COMER-like elements compared to the high variability of the ACME elements.

## Conclusion

In conclusion this work identifies a large composite SCC*mec* COMER-like element in the main clinical ST-BPH662 lineage of *S. epidermidis*. The exceptional stability of this large element supports the contribution of this element to the success of this important lineage of clinical *S. epidermidis* isolates.

## Data AvailabiliTy Statement

The datasets generated for this study can be found in the GenBank (accession numbers SAMN12840193–SAMN12840250).

## Author Contributions

NA performed the bulk of the laboratory and genomic work, and wrote the manuscript. RZ provided genomic training, contributed high throughput genomic data, and participated in the writing of the manuscript. CJ and CI were involved in phenotypic of clinical isolates and DNA extraction for genome sequencing. JR was involved in genome annotation and DNA extraction. AR was co-supervisor of NA and was involved in data analysis and manuscript preparation. IG supervised the clinical isolate collection and susceptibility testing. JM was co-supervisor of NA and was involved in data analysis and manuscript preparation. KH was involved in planning of the work, data analysis, and manuscript writing. MO conceived and supervised the work and contributed to the writing of the manuscript.

## Conflict of Interest

The authors declare that the research was conducted in the absence of any commercial or financial relationships that could be construed as a potential conflict of interest.

## Footnotes

1 www.rstudio.com/

2 www.bioinformatics.org/sms/rev_comp.html

3 Snapgene.com

4 https://www.socscistatistics.com
